# SARS-CoV-2-specific antibody responses following BNT162b2 vaccination in individuals with multiple sclerosis receiving different disease-modifying treatments

**DOI:** 10.3389/fneur.2023.1092999

**Published:** 2023-02-24

**Authors:** Anastasia Lambrianides, Elie Deeba, Maria Hadjiagapiou, Marios Pantzaris, George Krashias, Christina Christodoulou

**Affiliations:** ^1^Department of Neuroimmunology, The Cyprus Institute of Neurology and Genetics, Nicosia, Cyprus; ^2^Department of Molecular Virology, The Cyprus Institute of Neurology and Genetics, Nicosia, Cyprus; ^3^Postgraduate School, The Cyprus Institute of Neurology and Genetics, Nicosia, Cyprus

**Keywords:** SARS-CoV-2, multiple sclerosis, IgG antibodies, vaccines, disease-modifying treatments

## Abstract

**Introduction:**

The study aims to evaluate the concentration of IgG antibodies against the receptor-binding domain of the SARS-CoV-2 spike1 protein (S1RBD) in BNT162b2- vaccinated relapsing-remitting multiple sclerosis (RRMS) individuals receiving disease-modifying treatments (DMTs).

**Methods:**

Serum from 126 RRMS volunteers was collected 3 months after the administration of the second dose of the Pfizer-BioNTech BNT162b2 vaccine. Additional samples were analyzed after the administration of the booster dose in fingolimod- treated MS. Anti-S1RBD IgG antibody concentrations were quantified using the ABBOTT SARS-CoV-2 IgG II Quant assay.

**Results:**

Anti-S1RBD IgG antibody concentrations in RRMS individuals receiving natalizumab, interferons, teriflunomide, and dimethyl fumarate showed no significant difference to those in healthy controls. However, fingolimod-treated MS individuals showed a marked inability to produce SARS-CoV-2- specific antibodies (*p* < 0.0001). Furthermore, a booster dose was not able to elicit the production of IgG antibodies in a large portion of matched individuals.

**Discussion:**

A possible explanation for the altered immune response in fingolimod- treated MS individuals could be due to the medication inhibiting the circulation of lymphocytes, and possibly in turn inhibiting antibody production. Overall, patients on DMTs are generally of no disadvantage toward mounting an immune response against the vaccine. Nevertheless, further studies require evaluating non-humoral immunity against SARS-CoV-2 following vaccination, as well as the suitability of such vaccinations on patients treated with fingolimod.

## 1. Introduction

Severe acute respiratory syndrome coronavirus 2 (SARS-CoV-2), the causative agent for Coronavirus disease 2019 (COVID-19), has claimed over 6.5 million lives globally (October, 2022) (1). Vaccines that have received emergency approval for human use by the food and drug administration (FDA) or European medicines agency (EMA) include those from Pfizer-BioNTech, Moderna, AstraZeneca, and Janssen (2, 3). All the above vaccines have gone through clinical trials where their safety and efficacy were evaluated in previously healthy individuals (4). Of equal importance, there are no diseases, other than history of severe allergic reactions toward vaccinations, that are considered as contraindications for the use of these vaccines in the general population. Nevertheless, it remains to be seen whether the already approved vaccines are effective at inducing an adequate immune response in vaccinated individuals with different chronic neurological diseases, especially those with multiple sclerosis (MS) receiving different disease- modifying treatments (DMTs). Obtaining such information is of primary importance since it would highlight the suitability of the above vaccines for these individuals. This information can be utilized in the clinic by the treating physician for the benefit of the patients.

The Cyprus Institute of Neurology and Genetics (CING), as the reference center for neurological diseases in the Republic of Cyprus, treats patients with a wide range of neurological diseases. Following the guidelines of the WHO, the majority of these patients have been vaccinated against SARS-CoV-2. Interestingly, these patients are also treated with different immunomodulatory or immunosuppressive therapies. The effect of these therapies on the already approved Pfizer-BioNTech's BNT162b2 SARS-CoV-2 vaccines requires exploration to decide whether administration of booster doses would be beneficial.

The current study aims to evaluate for the first time the levels of antibodies against the receptor-binding domain of the SARS-CoV-2 spike1 protein (S1RBD) in BNT162b2-vaccinated MS individuals receiving different DMTs [natalizumab, fingolimod, teriflunomide, dimethyl fumarate, interferon β-1a (IFN β-1a), and interferon β-1b (IFN β-1b)].

## 2. Materials and methods

### 2.1. Ethical approval and subject recruitment

This study was approved by the Cyprus National Bioethics Committee (EEBK/EΠ/2020/23). All participants completed and signed an informed consent form.

### 2.2. Study population and sample collection/processing

A total of 126 volunteers with clinically definite relapsing-remitting MS and 52 healthy volunteers (HC) signed up for the study. Blood samples were collected from MS volunteers upon request from the Neuroimmunology department at The Cyprus Institute of Neurology and Genetics. The average number of days from the second dose to the booster dose was 90 days as indicated by the Ministry of Health in Cyprus. Throughout the study, patients that had COVID confirmed with PCR testing were excluded. In more detail, the inclusion criteria were: (1) patients above 18 years of age; (2) patients with clinically definite multiple sclerosis (CDMS) with clear clinical course of relapsing-remitting; (3) patients not experiencing any relapse symptoms during blood collection; (4) availability of a detailed clinical history [age of onset, disease duration calculated as the duration between sample acquisition and age of onset, Expanded Disability Status Scale (EDSS) score obtained on the day of sample acquisition, and treatments received]; and (5) being born in Cyprus and have resided in Cyprus from birth to at least early adult life. Exclusion criteria were: (1) presence of relapse in the 30 days before enrolment in the study; (2) inability or unwillingness to provide informed consent; (3) a history of alcohol or drug abuse; (4) pregnancy; and (5) history of previous SARS-CoV-2 infection. The inclusion and exclusion criteria, that are not solely MS-related, can be similarly extended to the healthy control group, save for the addition of an exclusion criterion that an individual may have any neurodegenerative, autoimmune, or underlying health issues. Table 1 shows the demographic details and clinical characteristics (EDSS, diseases duration, treatment at time of blood collection) of the MS volunteers and HCs. Other relevant data collected included SARS-CoV-2 infection history and lymphocyte counts for MS volunteers receiving fingolimod.

**Table 1:** Demographic and clinical characteristics of MS and healthy volunteers.

**Features**	**MS group (*n* = 126)**	**HC group (*n* = 52)**	***p*-value**
Age [mean (SD)]	45.08 ± 9.33	46.67 ± 12.86	0.340
Sex (male/female)	31/95	21/31	**0.046**
Duration of disease in years [median (interquartile range)]	9 (5–16)	N/A	
EDSS [median (interquartile range)]	3 (2–3.5)		
Type of treatment [*n* (%)]		N/A	
IFNβ-1a	42 (33.3%)		
Fingolimod	34 (27%)		
Natalizumab	26 (20.6%)		
Dimethyl fumarate	11 (8.7%)		
Teriflunomide	7 (5.6%)		
IFNβ-1b	6 (4.8%)		

The Mann-Whitney U-test was used for age matching, and the Fisher's exact test was used for sex matching.

MS, Multiple sclerosis; HCs, Healthy volunteers; RR, Relapsing Remitting MS; SP, Secondary Progressive MS; PP, Primary Progressive MS; IFN, Interferon; SD, Standard Deviation; N/A, Not Applicable. Bold value shows statistical significance.

The timing of vaccinations followed the guidelines set by the EMA and the protocol set by the Ministry of Health in Cyprus, where the second dose was administered 3 weeks after the initial dose of BNT162b2 and the booster dose administered 3 months after the second dose. Blood samples were collected from all volunteers 3 months after the second vaccination dose. Reviewing preliminary results warranted additional analysis from a select MS group, as such MS volunteers receiving fingolimod were asked to return for another blood sample at least 2 weeks after receiving the booster dose. Note that due to the volunteering nature of the study, some volunteers were not willing to further donate blood. Additionally, due to volunteers getting infected with SARS-CoV-2 during the time between vaccination doses, a follow-up sample was not suitable for the purpose of the study.

Blood samples were collected in tubes containing clotting activators at the COVID-19 sampling unit of The Cyprus Institute of Neurology and Genetics. Following blood collection, samples were centrifuged for 10 min at 500 × g at 20°C to obtain cell-free serum. Serum was stored at −20°C until analysis.

### 2.3. Anti-S1RBD IgG quantification analysis

Part of the serum obtained from the two groups of the study was used to quantify the level of Anti-S1RBD IgG antibodies. The quantification was performed using the ABBOTT SARS-CoV-2 IgG II Quant assay (REF# 6S60-22) on an ABBOTT ARCHITECT i1000SR instrument. The assay is an automated, two-step chemiluminescent microparticle immunoassay used for qualitative and quantitative determination of IgG antibodies against S1RBD of the SARS-CoV-2 from human serum and plasma. The SARS-CoV-2 IgG II Quant calibrator package (REF# 6S60-02) and the SARS-CoV-2 IgG II Quant control package (REF# 6S60-12) were run on the instrument prior to sample analysis. According to the manufacturer, the cut-off is set at 50.0 AU/mL, and the analytical measuring interval is set between 21.0 (limit of quantification) and 40,000.0 AU/mL (upper limit of quantification). Additional information on performance characteristics of the assay can be found in the manufacturer's manual. Based on the recommendations of the National Institute of Biological Standards and Control (NIBSC) and WHO, the concentrations were converted into Binding antibody units per mL (BAU/mL) through multiplying AU/mL by a factor of 0.142. The corresponding cut-off value becomes 7.1 BAU/mL.

### 2.4. Statistical analysis

The Mann-Whitney *U*-test and Fisher's exact test were used for age- and sex- matching, respectively. The Mann-Whitney *U*-test was used to evaluate significance in the differences between antibody levels in different groups. Simple linear regression and point-biserial correlation were used to analyze the correlation between antibody levels and lymphocyte count. The GraphPad Prism v8·00 for Windows software program was used to perform the statistical analyses (GraphPad Software, La Jolla, California, USA).

## 3. Results

### 3.1. Anti-S1RBD IgG antibody concentrations in MS and HC volunteers

**Figure 1:**
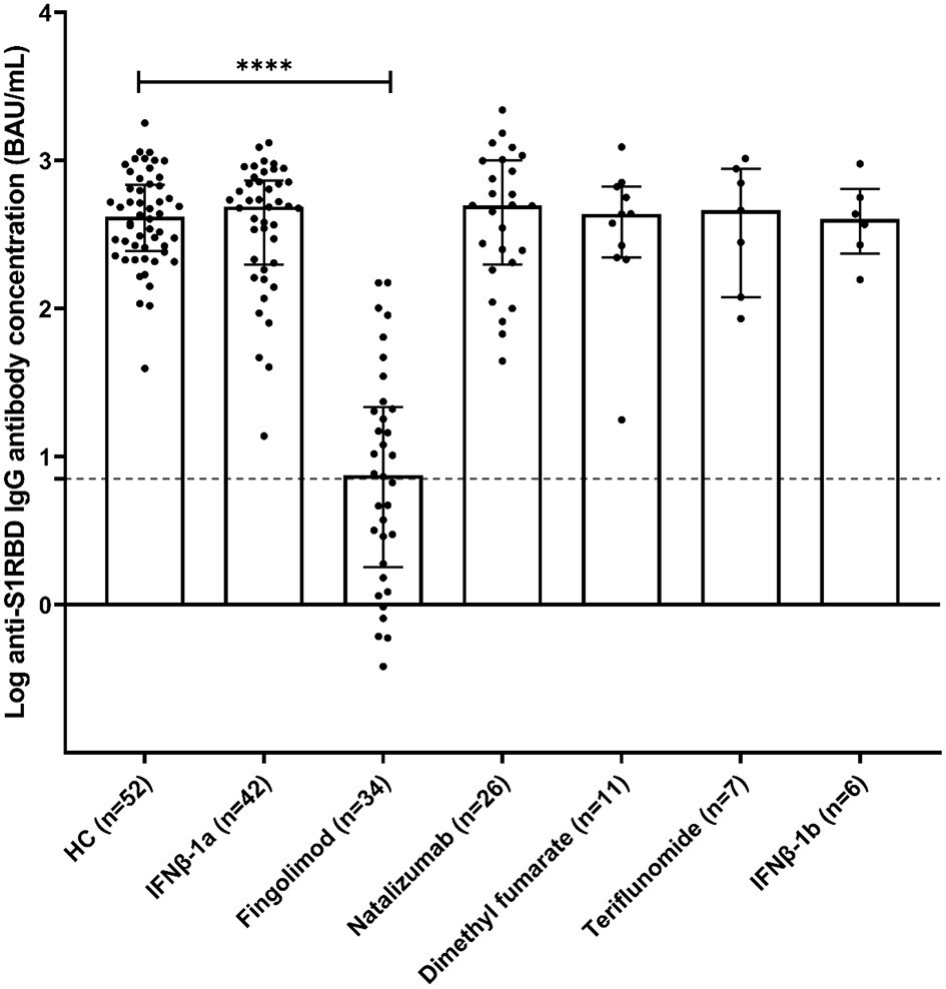
Anti-S1RBD IgG antibody levels in healthy volunteers (HC) and MS volunteers receiving various DMTs [interferonβ-1a (IFNβ-1a), interferonβ-1b (IFNβ-1b), natalizumab, fingolimod, dimethyl fumarate, and teriflunomide]. Bars represent median and interquartile ranges. The dotted line represents the cut-off value (7.1 BAU/mL). ****p < 0.0001.

Three months after the second vaccination dose, all of the HC group were found positive for anti-S1RBD IgG antibodies at a median (interquartile range) of 415.6 BAU/mL (244.9–686.5). Similarly, MS individuals receiving different medications were found to be positive for anti-S1RBD IgG antibodies, as well as comparable to the HC group, with medians (interquartile range) of 487.3 BAU/mL (197.8–730.6) for MS_IFNβ−1*a*_, 495.3 BAU/mL (199.1–999.5) for MS_Natalizumab_, 434.4 BAU/mL (220.9–663.8) for MS_Dimethylfumarate_, 460.4 BAU/mL (119.5–878.5) for MS_Teriflunomide_, and 402.4 BAU/mL (240.6–660.1) for MS_IFNβ−1*b*_. On the other hand, around half of the MS individuals receiving fingolimod (18/34; 52.9%) were positive for anti-S1RBD IgG antibodies with a significantly lower concentration [median (interquartile range); 7.5 BAU/mL (1.8–21.6)] compared to the HC group (*p* < 0.0001; Figure 1).

### 3.2. Anti-S1RBD IgG antibody level vs. lymphocyte count in fingolimod-treated MS individuals

**Figure 2:**
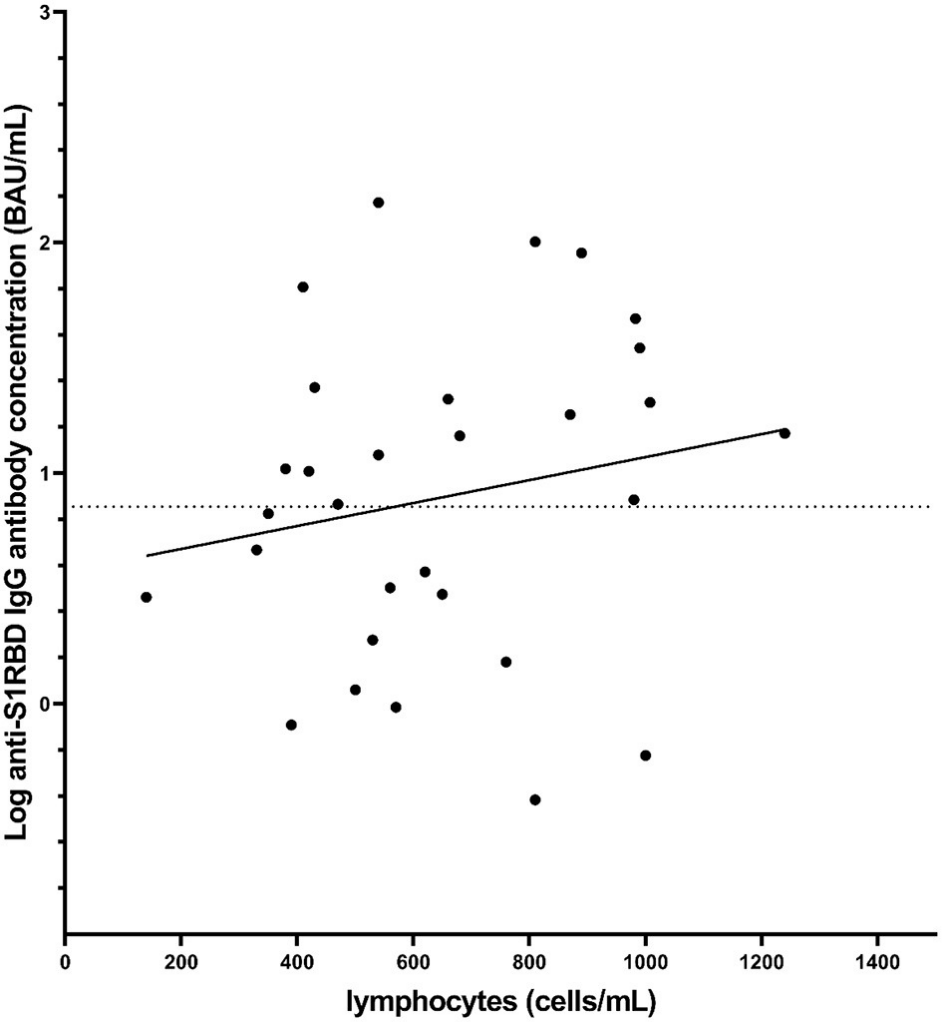
Distribution of anti-S1RBD IgG antibody levels measured after the second vaccination dose as a function of lymphocyte count also measured after the second vaccination dose in MS volunteers receiving fingolimod (*n* = 30). The dotted line represents the cut-off value (7.1 BAU/mL). The solid line represents a best fit line based on simple linear regression (*p* = 0.45).

Further analysis focused on the MS_Fingolimod_ group, where lymphocyte count data was collected for 30 individuals and measured independently by their physician around 4 weeks after their second vaccination dose. There was no significant correlation between lymphocyte counts and the concentration of anti-S1RBD IgG antibodies (linear regression; *p* = 0.45, point-biserial correlation; *p* = 0.08; *r* = 0.33; 95% CI = −0.04–0.61) (Figure 2). We note that, although there was no significance, there seems to be a trend showing higher anti-S1RBD IgG antibody concentrations with higher lymphocyte counts (correlation coefficient *r* > 0).

### 3.3. Change in antibody level following booster dose in fingolimod-treated MS individuals

**Figure 3:**
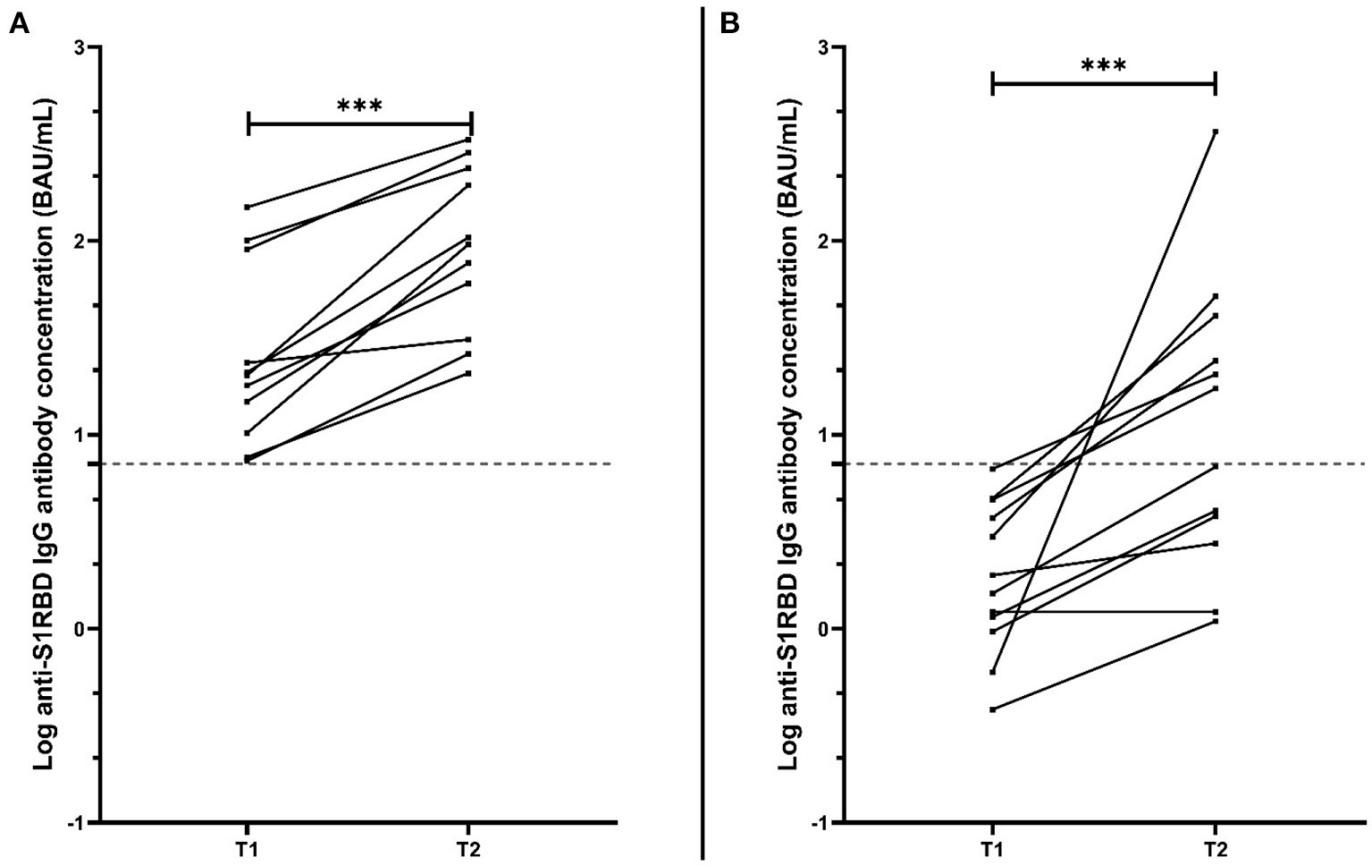
Anti-S1RBD IgG antibody level comparison between matched MS volunteers receiving fingolimod at 2 different time points: T1, 3 months after the second vaccination dose; and T2, at least 2 weeks after the booster dose. **(A)** Represents only MS_Fingolimod_ volunteers who were found positive for anti-S1RBD IgG at T1 (*n* = 11), while **(B)** represents those who were found negative for anti-S1RBD IgG at T1 (*n* = 12). The dotted line represents the cut-off value (7.1 BAU/mL). ***p < 0.001.

Based on the low concentrations of anti-S1RBD IgG antibodies measured in MS_Fingolimod_, as well as the recommendations for a SARS-CoV-2 booster dose administration, a follow-up sample was taken from MS_Fingolimod_ volunteers at least 2 weeks after the booster dose (T2) [median (interquartile range); 4.9 weeks (3.4–5.5)]. Anti-S1RBD IgG antibody levels were measured for 26 MS_Fingolimod_, of which 11 were previously found positive 3 months after the second vaccination dose (T1), and 12 were previously found negative at T1. After the booster dose, there was a significant increase in antibody concentration in MS_Fingolimod_ previously found positive at T1 from 20.3 BAU/mL (10.2–90.1) to 96.1 BAU/mL (30.9–236.8) (*p* < 0·001; Figure 3A). Similarly, antibody levels in MS_Fingolimod_ previously found negative at T1 significantly increased at T2 to a median (interquartile range) of 12.1 BAU/mL (3.0–36.9) (*p* < 0.001; Figure 3B), with half of those remaining negative after the booster dose. Analysis comparing antibody levels with lymphocyte count after the booster dose showed that the trend shown above appears to hold true, however without reaching significance (linear regression; *p* = 0.64, point-biserial correlation; *p* = 0.46; *r* = 0.15; 95% CI = −0.26–0.52) (graph not shown).

## 4. Discussion

With the seemingly unstoppable spread of SARS-CoV-2, and its variants, there was a need to ensure the safety of individuals with underlying comorbidities, specifically immunocompromised individuals. The neuroimmunology department at CING accepts and oversees the treatment of hundreds of individuals with MS in the Republic of Cyprus. Therefore, we aimed to understand the effect of different DMTs received by MS individuals on the levels of anti-S1RBD IgG antibodies produced after the full vaccination regimen with BNT162b2.

With the exception of one DMT (fingolimod), we did not observe a significant effect of different MS- directed medications on the ability of the immune system to produce anti-S1RBD antibodies against the full course of BNT162b2 vaccination regimen. Other studies have reported similar findings (5–10). However, we point out some discrepancies found between our results and results from Pitzalis et al. (8), whereby their results showed a significantly lower level of antibodies produced in MS treated with teriflunomide and natalizumab compared to the healthy control group. Such a discrepancy could be attributed to our small sample size for the two treatment groups, as well as large range in the antibody levels given the small sample sizes. Hence, we note the importance of unifying global data to further understand the effect of different medications in such niche groups. Our focus then turned to MS volunteers receiving fingolimod where, similar to other reports (5–12), we found significantly lower antibody levels compared to other MS and healthy volunteers. More so, such results were not exclusive to the type of vaccine used but were also observed in MS individuals vaccinated with Oxford-AstraZeneca's ChAdOx1-S (12) and Sinovac's CoronaVac vaccine (13). We can, therefore, further confirm a SARS-CoV-2- specific humoral immune response impairment due to treatment with fingolimod.

Due to the aggressive mode of action of fingolimod, we explored the possible relationship between circulating lymphocyte count and antibody production. Although our results showed a positive trend, i.e., higher lymphocyte counts correlate with higher antibody levels, our analysis did not return significance, possibly due to the low sample size. Nonetheless, this trend was also reported in different studies (7, 10, 14), where both B- and T- cell responses were measured and it was shown that there is a marked immunological impairment in MS individuals treated with fingolimod compared to those treated with natalizumab (7) or IFNβ (10), leading to the limited anti-S antibody production and T-cell activation.

In an effort to continue monitoring the SARS-CoV-2- specific humoral immune response in MS individuals receiving fingolimod, their anti-S1RBD IgG antibody levels were measured again after the administration of the BNT162b2 booster dose. Our results show that the booster shot was able to induce a significant increase in antibody levels. We also note that, of those who had tested negative for antibodies after their second vaccination dose, fifty percent converted to seropositive for anti-S1RBD IgG antibodies. The low number of seroconversions in MS individuals treated with fingolimod following a booster dose has also been observed in other studies as summarized in Table 2. Other studies by König et al. and Idda et al. do not point out changes in seroconversion, but rather report significantly lower concentrations and/or significantly reduced immunity compared to healthy vaccinated individuals (19, 20). Though these findings do not contradict with our results, they are not directly comparable with our study and therefore could not be included in the table, however we can note that both studies recruit <50 patients treated with fingolimod. Given that these observational studies, similar to this study, recruit a limited sample size, a larger cohort would be needed to confirm and further clarify the effect of DMTs on booster vaccinations, possibly achieved through international collaboration. In terms of the correlation between antibody level and lymphocyte count, our results show a similar trend before and after the booster dose, suggesting that additional vaccine administration might not be as effective if the lymphocyte count is low in fingolimod- treated individuals. Indeed, this conclusion was also inferred in another study that showed discontinuation of fingolimod treatment is significantly correlated with antibody production following booster dose administration (21). We note that natural immunization by SARS-CoV-2 infection, although beneficial for the immunity of the patients, does not interfere with the interpretation of our results, given the aim of the study at analyzing the changes in the levels of antibodies between vaccination doses.

**Table 2:** Comparison between different studies reporting seroconversion of fingolimod-treated MS patients following SARS-CoV-2 booster dose.

**Authors**	**Vaccine used**	**Total patients recruited**	**Antibody-negative patients after second dose**	**Seroconverted patients following booster dose**	**References**
Achiron et al.	BNT162b2	10	10	2	(14)
Achtnichts et al.	BNT162b2, mRNA-1273	8	8	4	(15)
Maglione et al.	BNT162b2, mRNA-1273	13	11	7	(16)
Meyer-Arndt et al.	BNT162b2, mRNA-1273	29	25	9	(17)
Tallantyre et al.	BNT162b2, mRNA-1273	15	15	7	(18)

This study has several limitations. Due to low turnout of volunteers, one limitation of the study was sample size, which had restricted the data to a handful of DMTs. Nonetheless, the results and trends shown in this study are consistent with other studies on the topic. Additionally, we were also restricted to the type of vaccine studied, as other types (ChAdOx1-S and mRNA-1273) were not administered in the Republic of Cyprus, in enough numbers to warrant meaningful analysis. The participant dropout after the booster dose led to an even more restricted sample size, which means that such results should be approached with caution and not be considered as wholly representative. Other limitations include the uncertainty of SARS-CoV-2 infection in both the MS and the HC groups. SARS-CoV-2 history was based solely on patient/control declaration and anti-nucleocapsid antibodies have not been checked for asymptomatic events, however, since the purpose of the study was to assess the levels of antibodies against the receptor-binding domain of the SARS-CoV-2 spike1 protein (S1RBD) in BNT162b2-vaccinated MS individuals receiving different DMTs, it is unlikely it would have not affected the comparison. Another limitation is the lack of Indirect information on T cell responses, which would have enabled us to get a more complete picture of a patient's immune status, by using additional tests that measure the presence and function of specific types of immune cells, such as CD4 T lymphocytes and cytokines such as IFN. These tests can indeed provide important information about how the immune system is responding to infection with COVID-19. However, it is important to note that the interpretation of these results can be complex. Future studies could follow the data on a larger longitudinal scale, while also incorporating data on T cell- based responses which might play a larger role in immunity against SARS-CoV-2. Additionally, future studies could focus on understanding the exact mechanism of fingolimod in terms of antibody production, by measuring the relationship between antibody levels and each lymphocyte subset.

## 5. Conclusions

The current study aimed to evaluate the IgG antibody levels against S1RBD of SARS-CoV-2 in BNT162b2-vaccinated Cypriot MS individuals receiving different DMTs. We showed that BNT162b2 was effective at inducing a sufficient humoral response comparable to healthy individuals, regardless of treatments received. However, the vaccine was unable to elicit the same response in fingolimod- treated MS individuals, where antibody levels, if positive, were significantly lower compared to those in MS individuals receiving other DMTs. Even with a booster dose, some MS individuals receiving fingolimod were not able to produce anti-S1RBD IgG antibodies, this could be attributed to the aggressive mode of action of fingolimod which effectively inhibits the immune system's ability to elicit any significant humoral responses toward an infection. Our results may aid the global effort in understanding antibody kinetics across different individuals receiving immunomodulatory medications. This may also help in better informing public health policies regarding vaccine efficacy and humoral immunity in immunocompromised individuals, as well as vaccine considerations against new emerging variants of concern.

## Data availability statement

The original contributions presented in the study are included in the article/supplementary material, further inquiries can be directed to the corresponding author.

## Ethics statement

The studies involving human participants were reviewed and approved by Cyprus National Bioethics Committee. The patients/participants provided their written informed consent to participate in this study.

## Author contributions

Conceptualization: CC, GK, and MP. Project administration: CC and MP. Data collection and curation: ED, AL, and MH. Formal analysis and interpretation: ED and AL. Funding acquisition: CC and MP. Methodology: ED and GK. Validation: ED, GK, and CC. Writing—original draft: ED. Writing—review and editing: AL, GK, and CC. All authors contributed to the article and approved the submitted version.
